# Racial Disparities in Transcatheter Edge-to-Edge Repair Versus Open Mitral Valve Surgery: A Nationwide Analysis

**DOI:** 10.14740/cr2226

**Published:** 2026-07-17

**Authors:** Ali Awad, Qusai Al Qudah, Joud Fahed, Mariam Chalhoub, M. Chadi Alraies

**Affiliations:** a Department of Internal Medicine, Detroit Medical Center, Wayne State University, Detroit, MI, USA; b Department of Medicine, University of Central Florida College of Medicine, Orlando, FL, USA; c Department of Medicine, Ascension Saint Agnes Hospital, Baltimore, MD, USA; d Department of Medicine, Case Western Reserve University, University Hospitals Cleveland Medical Center, Cleveland, OH, USA; e Department of Cardiovascular Medicine, Detroit Medical Center, Wayne State University, Detroit, MI, USA

**Keywords:** Racial differences, Procedural selection, Outcomes, TEER, Open mitral valve surgery

## Abstract

**Background:**

Racial disparities have been documented across multiple domains of cardiovascular care, yet data on mitral valve intervention remain limited. We examined racial differences in procedural selection and outcomes following transcatheter edge-to-edge repair (TEER) and open mitral valve surgery.

**Methods:**

Using the National Inpatient Sample (NIS, 2016–2022), we identified 26,456 adults undergoing mitral valve intervention. Multivariable regression models were used to assess racial differences in mortality, procedure type, length of stay, and charges, adjusting for demographics, comorbidities, hospital characteristics, and illness severity.

**Results:**

TEER comprised 89% of procedures. Black patients were younger (mean 62 vs 70 years) but presented with higher illness severity. After adjustment, Black patients had greater odds of receiving TEER (odds ratio (OR) = 1.36; 95% confidence interval (CI), 1.15–1.61). Mortality disparities persisted after adjustment: Black patients (OR = 1.49; 95% CI, 1.01–2.19; P = 0.045) and Asian/Pacific Islander patients OR = 2.20; 95% CI, 1.30–3.72; P = 0.003). Late presentation was more common among Black (24%) and Hispanic (27%) compared with White patients (13%).

**Conclusions:**

Black and Asian/Pacific Islander patients experience elevated inpatient mortality following mitral valve intervention that is not fully explained by covariates available in NIS. Late referral patterns are consistent with barriers to timely specialized care in minority populations.

## Introduction

Mitral regurgitation (MR) affects over 4 million Americans and is the most common valvular heart disease in developed countries [1, 2]. Nearly half of patients with severe symptomatic disease do not receive surgical intervention, often due to perceived operative risk [3, 4]. Transcatheter edge-to-edge repair (TEER) offers a minimally invasive alternative. The COAPT trial demonstrated sustained reductions in heart failure hospitalizations and mortality at 5 years among selected patients with secondary MR [5, 6]. MITRA-FR showed more modest results, underscoring the importance of patient selection [7]. The EVEREST II trial established the feasibility of percutaneous repair [8]. TEER adoption has expanded substantially, but whether minority patients have benefited equitably is unknown.

Racial disparities in cardiovascular care are well documented [9, 10]. Black patients face higher mortality after coronary bypass and worse heart failure outcomes. Studies of transcatheter aortic valve replacement (TAVR) show that Black and Hispanic patients are underrepresented yet have comparable or better outcomes when they do receive treatment [11–14]. Data on racial disparities in mitral valve intervention remain sparse, and existing literature predates widespread TEER adoption.

Given that Black and Hispanic patients bear a disproportionate burden of heart failure and MR, understanding whether racial disparities exist in this domain has clinical and policy implications. Prior work has not examined TEER utilization patterns by race, whether outcomes differ after accounting for baseline risk, or what mechanisms might contribute to any observed differences.

We conducted a nationwide analysis to evaluate racial differences in the utilization of TEER versus open mitral valve surgery, assess disparities in inpatient mortality, length of stay, and hospital charges, and explore referral patterns as a potential underlying mechanism.

## Materials and Methods

### Data source and study population

We analyzed the National Inpatient Sample (NIS) from 2016 through 2022. We identified adults aged ≥ 18 years undergoing mitral valve intervention using International Classification of Diseases, 10th Revision, Procedure Coding System (ICD-10-PCS) codes. TEER was defined by code 02UG3JZ (Supplement Mitral Valve with Synthetic Substitute, Percutaneous Approach). Open mitral valve surgery included both repair codes (02QG0ZZ, 02QG3ZZ, 02QG4ZZ) and replacement codes (02RG07Z, 02RG08Z, 02RG0JZ, 02RG0KZ, 02UG0JZ, 02UG4JZ). Patients who underwent both TEER and open surgery during the same hospitalization were excluded.

Race/ethnicity was ascertained from the NIS RACE variable, which combines information on race and Hispanic ethnicity from state inpatient databases. We categorized patients as White, Black, Hispanic, or Asian/Pacific Islander. Records coded as “other” or with missing race/ethnicity were excluded to permit direct comparisons among the four principal groups. The final analytic cohort comprised 26,456 hospitalizations, corresponding to approximately 132,280 weighted discharges.

### Outcomes and covariates

The primary outcome was inpatient mortality. Secondary outcomes included procedure type (TEER vs open surgery), length of stay, total hospital charges, and late presentation. We defined late presentation as any of: nonelective admission, emergency department (ED) presentation, or transfer from another facility. These markers indicate patients who did not receive elective, planned intervention through outpatient referral pathways.

Covariates included age, sex, primary payer, median household income quartile by patient ZIP code, hospital characteristics (bed size, teaching status, census region), and comorbidities identified via International Classification of Diseases, 10th Revision, Clinical Modification (ICD-10-CM) diagnosis codes: hypertension, diabetes mellitus, heart failure, atrial fibrillation, coronary artery disease, chronic kidney disease, chronic obstructive pulmonary disease, cardiomyopathy, pulmonary hypertension, and obesity. All comorbidities were ascertained by scanning the I10_DX1–I10_DX40 diagnosis fields against published ICD-10-CM code ranges. Illness severity was quantified using the All Patient Refined Diagnosis Related Group (APR-DRG) severity of illness subclass, an ordinal variable ranging from 1 (minor) to 4 (extreme). In regression models, APR-DRG severity was entered as a continuous covariate; we also constructed a normalized severity score (APR-DRG severity divided by 4) for descriptive purposes. Severe heart failure, defined as a heart-failure diagnosis with APR-DRG severity ≥ 3, was used as a clinical proxy for likely secondary (functional) MR in stratified analyses.

### Statistical analysis

All analyses incorporated survey procedures to account for the complex sampling design of the NIS. We constructed sequential multivariable models: model 1 (unadjusted); model 2 (demographics: age, sex, payer, income quartile, year); model 3 (model 2 + hospital characteristics); model 4 (model 3 + comorbidities); model 5 (model 4 + procedure type and APR-DRG severity). This approach allows identification of which covariate sets attenuate observed disparities.

Binary outcomes were analyzed using logistic regression, yielding odds ratios (ORs) with 95% confidence intervals. Length of stay was modeled using negative binomial regression. Hospital charges were analyzed using generalized linear models with gamma distribution and log link. We conducted sensitivity analyses restricting to large-bedsize hospitals, restricting to teaching hospitals, stratified by severe heart failure status, and using race-as-treatment inverse probability of treatment weighting (IPTW). To estimate the association of race with outcomes after balancing measured confounders, we fit a logistic-regression propensity score predicting Black (vs White) race conditional on age, sex, insurance, income quartile, hospital characteristics, comorbidities, and APR-DRG severity. Inverse probability of treatment weights were calculated as Black/propensity score (PS) + (1 − Black)/(1 − PS). Balance was assessed using standardized mean differences (SMDs) before and after weighting; we considered |SMD| ≤ 0.10 as adequate balance. Effective sample size was computed as (Σw)^2^/Σw^2^. Temporal trends were assessed using race-by-year interaction terms. We used complete-case analysis for covariates in multivariable models. All tests were two-sided with α = 0.05. Analyses were performed using Stata 19 (StataCorp, College Station, TX).

### Ethics statement

This study was reviewed by the Wayne State University Institutional Review Board (IRB) and was determined to be exempt from full review because it used fully deidentified data from the Healthcare Cost and Utilization Project (HCUP) National Inpatient Sample (NIS), a publicly available administrative database (IRB protocol number: 20-01-1719). The HCUP Data Use Agreement was completed by the investigators before data access. Under the Common Rule, research using deidentified NIS data is generally exempt from IRB approval. The study did not involve direct interaction with human participants, or identifiable private information, and was conducted in accordance with the ethical principles of the 1964 Declaration of Helsinki and its later amendments.

## Results

### Study population

**Figure 1:**
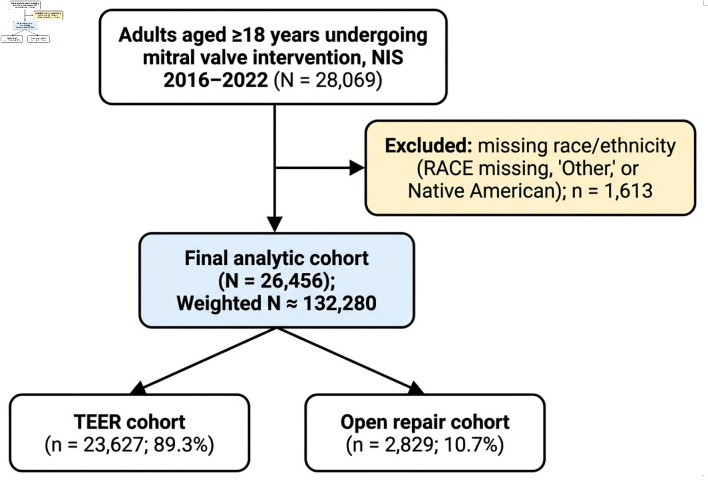
Study flow diagram. NIS: National Inpatient Sample; TEER: transcatheter edge-to-edge repair.

Figure 1 shows patient flow through the study. The final cohort included 26,456 hospitalizations (weighted n = 132,280). TEER accounted for 89% of procedures, and open surgery for 11%. The racial distribution was 84% White, 8% Black, 5% Hispanic, and 3% Asian/Pacific Islander.

### Baseline characteristics

Table 1 presents baseline characteristics by race. Black patients were younger than White patients (mean 62 vs 70 years, P < 0.001) and more often female (53% vs 41%). Socioeconomic differences were pronounced: 45% of Black patients resided in the lowest income quartile versus 17% of White patients, and Medicaid coverage was more common among Black (14%) versus White (3%) patients. Hypertension (88% vs 72%), diabetes (29% vs 16%), and chronic kidney disease (41% vs 22%) were more prevalent among Black patients, as were heart failure (76% vs 59%) and cardiomyopathy (37% vs 15%). Severe heart failure (heart failure with APR-DRG severity ≥ 3, used as a proxy for likely secondary MR) was substantially more prevalent in Black patients (47% vs 29% in Whites, P < 0.001). The mean normalized severity score was higher among Black patients (0.66 vs 0.60, P < 0.001), indicating greater illness severity at presentation.

**Table 1:** Baseline Characteristics by Race/Ethnicity

Characteristic	Overall (n = 26,456)	White (n = 22,050)	Black (n = 2,178)	Hispanic (n = 1,382)	Asian/PI (n = 846)	P
Age, mean (years)	68.7	69.6	62.2	66.4	64.3	< 0.001
Age < 50, %	8.8	7.4	17.8	13.2	16.6	< 0.001
Age 50–64, %	26.1	24.8	36.0	28.6	30.3	
Age 65–74, %	27.0	27.4	25.3	24.2	25.7	
Age 75–84, %	26.8	28.2	16.8	23.4	20.0	
Age ≥ 85, %	11.3	12.2	4.1	10.6	7.6	
Male, %	57.8	59.1	47.2	54.6	56.2	< 0.001
Insurance						< 0.001
Medicare, %	61.7	63.6	52.9	55.9	44.7	
Medicaid, %	4.8	3.2	14.3	12.2	10.6	
Private, %	30.4	30.4	27.9	26.8	40.2	
Other/Self-pay, %	3.2	2.8	4.9	5.1	4.5	
Income quartile						< 0.001
Q1 (lowest), %	19.9	17.2	44.6	29.9	8.7	
Q4 (highest), %	29.5	30.9	13.3	19.5	52.5	
Large hospital, %	72.2	71.7	73.3	74.5	77.5	0.005
Teaching hospital, %	91.1	90.9	92.8	90.9	91.8	0.117
Region						< 0.001
Northeast, %	19.6	20.2	16.6	18.7	15.5	
Midwest, %	24.1	26.0	19.5	8.8	11.4	
South, %	33.9	32.3	53.2	40.4	16.1	
West, %	22.4	21.6	10.7	32.1	57.1	
Comorbidities (ICD-10 ascertained)						
Hypertension, %	73.8	72.1	87.7	79.6	72.9	< 0.001
Diabetes, %	18.1	15.8	29.4	31.5	25.2	< 0.001
Obesity, %	14.3	13.4	22.8	18.7	8.4	< 0.001
Chronic kidney disease, %	23.8	21.8	40.5	29.7	22.5	< 0.001
COPD, %	11.9	11.9	15.3	10.2	5.7	< 0.001
Atrial fibrillation, %	58.7	60.5	44.2	53.0	58.0	< 0.001
Heart failure, %	60.6	58.6	75.8	69.3	57.7	< 0.001
Coronary artery disease, %	42.3	42.1	42.2	47.3	38.3	< 0.001
Cardiomyopathy, %	17.3	15.0	36.7	23.3	17.9	< 0.001
Pulmonary hypertension, %	22.9	21.6	32.5	26.3	26.0	< 0.001
Severe heart failure^a^, %	31.4	29.1	47.1	41.2	32.9	< 0.001
Late presentation						
Late referral composite, %	15.1	13.4	23.8	27.4	16.1	< 0.001
Nonelective admission, %	14.8	13.1	23.6	27.3	16.1	< 0.001
ED arrival, %	4.8	4.2	9.4	8.2	3.7	< 0.001
TEER (vs open repair), %	89.3	89.4	90.3	88.5	86.9	0.051

^a^Severe heart failure was defined as a heart-failure diagnosis with APR-DRG severity ≥ 3 and was used as a proxy for likely secondary (functional) mitral regurgitation. PI: Pacific Islander; ICD-10: ICD-10-CM: International Classification of Diseases, 10th Revision; SD: standard deviation; TEER: transcatheter edge-to-edge repair; COPD: chronic obstructive pulmonary disease; ED: emergency department.

### Procedure utilization

Unadjusted TEER rates were similar across groups (range 87–90%, P = 0.051). After multivariable adjustment, however, Black patients had 36% greater odds of receiving TEER compared with White patients (OR, 1.36; 95% CI, 1.15–1.61; P < 0.001) (Table 2). Although this pattern is consistent with guideline-based risk stratification favoring TEER in higher-risk patients [15, 16], it could equally reflect a higher prevalence of secondary (functional) MR in this population, for which TEER is the dominant indication. NIS does not contain mitral-valve-imaging data and cannot distinguish primary from secondary MR.

**Table 2:** Odds of Receiving TEER vs Open Surgery by Race/Ethnicity

Race (vs White)	Model 1 unadjusted	Model 2 + demographics	Model 3 + hospital	Model 4 fully adjusted*
Black	1.10 (0.95–1.28) P = 0.200	1.53 (1.30–1.79) P < 0.001	1.53 (1.30–1.80) P < 0.001	1.36 (1.15–1.61) P < 0.001
Hispanic	0.92 (0.77–1.09) P = 0.323	1.07 (0.89–1.28) P = 0.476	1.12 (0.93–1.34) P = 0.241	1.04 (0.86–1.25) P = 0.699
Asian/PI	0.79 (0.63–0.98) P = 0.031	0.99 (0.79–1.24) P = 0.934	1.03 (0.82–1.30) P = 0.773	1.01 (0.81–1.27) P = 0.914

PI: Pacific Islander; TEER: transcatheter edge-to-edge repair.

### In-hospital mortality

**Figure 2:**
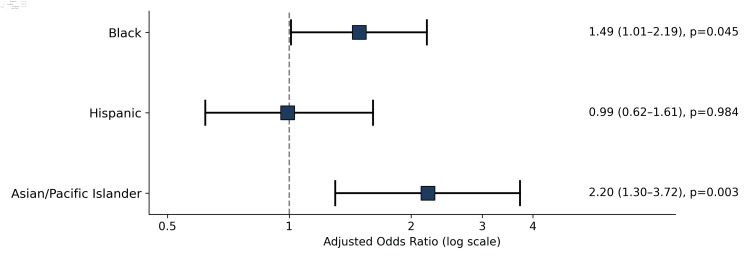
Forest plot showing adjusted odds ratios (ORs) with 95% confidence intervals for in-hospital mortality by race/ethnicity, with White patients as the reference group.

In-hospital mortality was overall low at 1.2% across the 26,456-patient cohort, consistent with contemporary national data on both TEER and surgical mitral repair. Within this low-event setting, unadjusted rates differed by race: White 1.1%, Black 2.0%, Hispanic 1.6%, Asian/Pacific Islander 2.1% (P < 0.001). In unadjusted models, Black patients had an OR of 1.85 (95% CI, 1.33–2.57) and Asian/Pacific Islander patients had an OR of 1.99 (95% CI, 1.20–3.29) compared with White patients. Sequential adjustment attenuated but did not eliminate these disparities. In fully adjusted models (Table 3), Black patients had an OR of 1.49 (95% CI, 1.01–2.19; P = 0.045) and Asian/Pacific Islander patients had an OR of 2.20 (95% CI, 1.30–3.72; P = 0.003). The Hispanic disparity was fully attenuated after adjustment (OR, 0.99; 95% CI, 0.62–1.61). Adjusted ORs for in-hospital mortality by race/ethnicity are shown in Figure 2.

**Table 3:** Odds of In-Hospital Mortality by Race/Ethnicity (Sequential Adjustment)

Race (vs White)	Model 1 unadjusted	Model 2 + demographics	Model 3 + hospital	Model 4 + comorbidities	Model 5 fully adjusted†
Black	1.85 (1.33–2.57) P < 0.001	1.89 (1.34–2.65) P < 0.001	1.80 (1.27–2.53) P = 0.001	1.42 (0.98–2.06) P = 0.060	1.49 (1.01–2.19) P = 0.045
Hispanic	1.48 (0.94–2.33) P = 0.088	1.50 (0.95–2.36) P = 0.083	1.43 (0.90–2.27) P = 0.127	1.36 (0.85–2.16) P = 0.199	0.99 (0.62–1.61) P = 0.984
Asian/PI	1.99 (1.20–3.29) P = 0.007	2.48 (1.48–4.16) P < 0.001	2.57 (1.51–4.37) P < 0.001	2.52 (1.47–4.33) P = 0.001	2.20 (1.30–3.72) P = 0.003

### Length of stay and hospital charges

Unadjusted mean length of stay was 7.8 days for Black, 7.2 for Hispanic, 6.1 for Asian/Pacific Islander, and 5.6 for White patients (P < 0.001). After adjustment, length of stay remained modestly elevated for Black (adjusted incidence rate ratio (IRR), 1.06; 95% CI, 1.01–1.12, P = 0.011) and Hispanic patients (adjusted IRR, 1.07; 95% CI, 1.01–1.14; P = 0.016), with Asian/Pacific Islander patients not significantly different from White patients (Table 4). Mean hospital charges ranged from $213,000 (White) to $293,000 (Hispanic; P < 0.001). Charge disparities persisted after adjustment: Black +7% (P = 0.002), Hispanic +19% (P < 0.001), and Asian/Pacific Islander +8% (P = 0.002) compared with White patients.

**Table 4:** Length of Stay and Hospital Charges by Race/Ethnicity

Race	Mean LOS (days)	Adjusted IRR (95% CI)	Mean charges ($)	Adjusted ratio (95% CI)	P value
White	5.6	Reference	212,715	Reference	—
Black	7.9	1.06 (1.01–1.12)	255,844	1.07 (1.02–1.11)	0.011/0.002
Hispanic	7.2	1.07 (1.01–1.14)	292,812	1.19 (1.14–1.25)	0.016/< 0.001
Asian/PI	6.4	1.03 (0.98–1.09)	283,508	1.08 (1.03–1.14)	0.274/0.002

PI: Pacific Islander; IRR: incidence rate ratio; LOS: length of stay; CI: confidence interval.

### Late presentation

Markers of late presentation were present in 15% of patients overall but varied by race: White 13%, Black 24%, Hispanic 27%, Asian/Pacific Islander 16% (P < 0.001). After adjustment, Black patients had an OR of 1.23 (95% CI, 1.08–1.41; P = 0.002) and Hispanic patients had an OR of 1.74 (95% CI, 1.47–2.06; P < 0.001) for the late-presentation composite compared with White patients (Table 5).

**Table 5:** Late or Emergent Presentation by Race/Ethnicity

Race	Late presentation (unadjusted %)	Composite OR (95% CI)	Nonelective OR (95% CI)	ED arrival OR (95% CI)
White	13%	Reference	Reference	Reference
Black	24%	1.23 (1.08–1.41) P = 0.002	1.25 (1.10–1.43) P = 0.001	1.26 (1.02–1.54) P = 0.031
Hispanic	27%	1.74 (1.47–2.06) P < 0.001	1.76 (1.48–2.08) P < 0.001	1.36 (1.06–1.75) P = 0.016
Asian/PI	16%	1.04 (0.85–1.27) P = 0.716	1.06 (0.86–1.29) P = 0.600	0.76 (0.50–1.15) P = 0.188

PI: Pacific Islander; CI: confidence interval; OR: odds ratio.

Decomposing the composite into its three constituents (nonelective admission, ED arrival, and inter-hospital transfer), Black patients had similar magnitudes of elevation in nonelective admission (adjusted OR, 1.25; 95% CI, 1.10–1.43) and ED arrival (adjusted OR, 1.26; 95% CI, 1.02–1.54) after adjustment for comorbidities and severity, with transfer-in too sparse for stable adjusted estimation. Hispanic patients showed the largest disparities in both nonelective admission (OR, 1.76; 95% CI, 1.48–2.08) and ED arrival (OR, 1.36; 95% CI 1.06–1.75).

**Figure 3:**
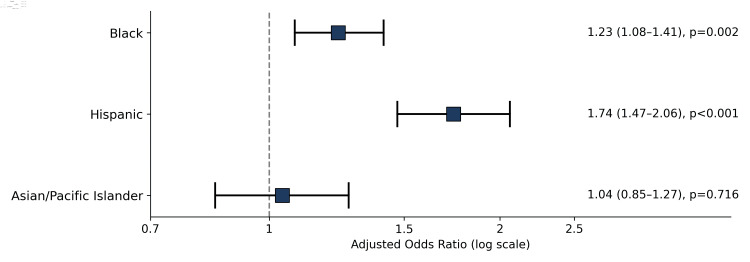
Forest plot of adjusted odds ratios (ORs) with 95% confidence intervals for late or emergent presentation by race/ethnicity, with White patients as the reference group.

Racial differences in late or emergent presentation are summarized in Figure 3.

### Sensitivity analyses

Mortality findings were robust in sensitivity analyses (Table 6). Restricting to large hospitals yielded a mortality OR of 1.72 (95% CI, 1.11–2.68; P = 0.016) for Black patients; restricting to teaching hospitals yielded an OR of 1.47 (95% CI 0.98–2.21; P = 0.065). Race-as-treatment IPTW (Black vs White, with balance verified at all |SMD| ≤ 0.10; effective sample size (ESS) = 1,802) attenuated the mortality disparity to non-significance (OR, 1.29; 95% CI, 0.63–2.61; P = 0.48), whereas the TEER selection disparity persisted (OR, 1.35; 95% CI, 1.05–1.73, P = 0.020). Race-by-year interaction terms were not significant for either mortality (P = 0.71) or TEER selection (P = 0.60), indicating no temporal change in disparities over the study period. Stratified analyses (Table 7) showed mortality disparities were more pronounced among TEER recipients than among open surgery patients.

**Table 6:** Sensitivity Analyses for In-Hospital Mortality

Analysis	Black OR (95% CI)	Asian/PI OR (95% CI)	N
Primary analysis (fully adjusted)	1.49 (1.01–2.19), P = 0.045	2.20 (1.30–3.72), P = 0.003	26,076
Large hospitals (bedsize 3) only	1.72 (1.11–2.68), P = 0.016	2.42 (1.36–4.30), P = 0.003	19,092
Teaching hospitals only	1.47 (0.98–2.21), P = 0.065	1.82 (1.04–3.21), P = 0.037	24,089
Severe heart failure subgroup	1.84 (1.21–2.78), P = 0.004	1.94 (1.05–3.60), P = 0.036	8,292
Non-severe heart failure subgroup	1.26 (0.48–3.27), P = 0.640	2.41 (0.92–6.31), P = 0.072	18,161
Race-as-treatment IPTW (Black vs White)	1.29 (0.63–2.61), P = 0.483	Not applicable	23,892

PI: Pacific Islander; CI: confidence interval; OR: odds ratio.

**Table 7:** Stratified Analyses by Procedure Type

Procedure	Race	Mortality, %	Adjusted OR (95% CI)	P value
TEER	White	1.0	Reference	—
	Black	1.9	1.56 (1.02–2.36)	0.038
	Hispanic	1.5	1.06 (0.64–1.76)	0.824
	Asian/PI	2.0	1.96 (1.09–3.52)	0.024
Open surgery	White	1.8	Reference	—
	Black	2.4	2.41 (0.98–5.95)	0.056
	Hispanic	2.1	1.56 (0.45–5.48)	0.485
	Asian/PI	2.6	2.29 (0.62–8.51)	0.216

TEER: transcatheter edge-to-edge repair; PI: Pacific Islander; CI: confidence interval; OR: odds ratio.

**Figure 4:**
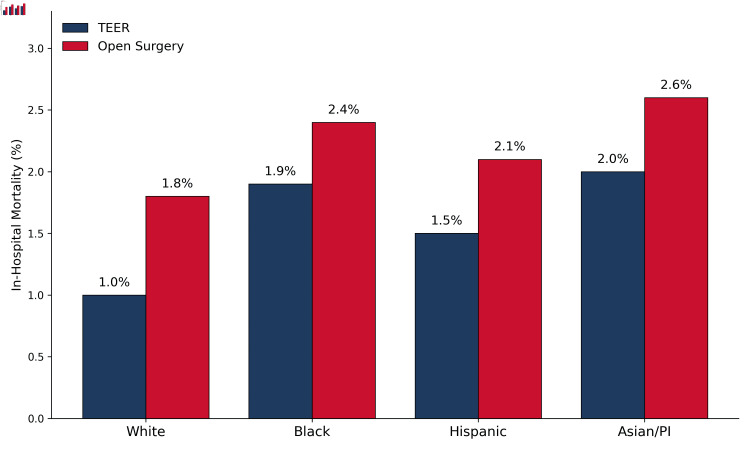
In-hospital mortality by race and procedure type. Bar plots comparing in-hospital mortality rates stratified by race/ethnicity for patients undergoing transcatheter edge-to-edge repair (TEER) and open mitral valve surgery. P values reflect adjusted comparisons between each racial group and White patients within each procedural stratum. PI: Pacific Islander.

Figure 4 displays in-hospital mortality stratified by race and procedure type, demonstrating that mortality disparities were more pronounced among TEER recipients than among open surgery patients.

## Discussion

In this nationwide study of over 26,000 mitral valve interventions, we found that Black and Asian/Pacific Islander patients experienced elevated inpatient mortality that persisted after adjustment for demographics, comorbidities, hospital factors, procedure type, and illness severity. Although absolute mortality was low across all groups (1.2% overall), the relative differences are clinically meaningful, given the volume of these procedures performed nationally each year. Black patients were more likely to receive TEER after adjustment, which may reflect risk-based selection, a higher prevalence of secondary MR, or both. However, minority patients more often presented late, suggesting barriers to timely specialized care.

These findings are consistent with prior literature on cardiovascular disparities [17–21]. Kyalwazi et al documented persistent Black–White gaps in cardiovascular mortality from 1999 to 2019, with disparities most pronounced among younger adults[17]. Glynn et al found widening heart failure mortality disparities, with Black women experiencing nearly threefold higher death rates [18]. In structural heart disease, Alkhouli et al reported that Black patients were underrepresented in TAVR but had comparable adjusted outcomes when treated [11]. Our data extend this literature to mitral valve intervention and show that even when minorities undergo treatment, outcomes differ.

Although NIS does not capture MR etiology directly, we used severe heart failure (heart-failure diagnosis with APR-DRG severity ≥ 3) as a clinical proxy for secondary (functional) MR. In this subgroup (31.4% of the cohort), Black patients had nearly twofold-higher in-hospital mortality (adjusted OR, 1.84; 95% CI, 1.21–2.78), whereas mortality among non-severe-heart failure patients did not differ significantly by race (adjusted OR, 1.26; 95% CI, 0.48–3.27). This pattern is consistent with secondary MR being independently associated with worse short-term outcomes, and with Black patients in our cohort having a markedly higher prevalence of severe heart failure (47% vs 29% in Whites, P < 0.001), suggesting that disease phenotype rather than race itself may drive much of the observed mortality difference.

The persistence of mortality disparities despite appropriate procedure selection is concerning. Possible explanations include unmeasured differences in disease severity or anatomy not captured by administrative codes, variation in procedural technique or institutional quality, differences in post-procedural care, or biological factors [22–24]. Rangrass et al showed that hospital quality explains more than half of the racial disparity in cardiac surgery mortality, raising the possibility that where patients receive care matters as much as whether they receive care [22].

The persistent Black–White disparity in TEER selection was robust to sensitivity restriction to large-volume and teaching hospitals (OR 1.33 and 1.38, respectively) and to race-balanced propensity-score weighting (OR 1.35), arguing against a pure hospital-availability explanation. However, several intersecting mechanisms remain plausible: Black patients in our cohort more frequently presented non-electively (adjusted OR 1.25) and through the ED (adjusted OR 1.26), and had substantially higher prevalence of severe heart failure (47% vs 29% in White patients), each of which may shift clinicians toward catheter-based therapy. We cannot, in administrative data, distinguish whether the residual procedure-selection disparity reflects clinical judgment about operative risk, patient preferences, or systemic factors that channel late-presenting patients toward less invasive therapies.

Multiple non-mutually exclusive mechanisms may contribute to the procedural disparity. First, Black patients in our cohort had markedly higher prevalence of severe heart failure (47% vs 29% in Whites), cardiomyopathy (37% vs 15%), and chronic kidney disease (41% vs 22%), a constellation that elevates operative risk and frequently shifts clinical decision-making toward TEER. Second, Black patients more often presented through non-elective channels (24% vs 13%), where staged surgical workup may be impractical and catheter-based intervention more feasible. Third, despite being younger on average (mean 62 vs 70 years), Black patients in our cohort had higher APR-DRG severity, consistent with the established “weathering” pattern of earlier-age but higher-severity cardiovascular disease in Black patients. We cannot adjudicate the relative contribution of these factors in administrative data, but the consistency of the disparity across multiple sensitivity analyses argues that it is not explained by any single mechanism.

Late presentation emerged as a potential mechanism. Nearly one quarter of Black patients and over one quarter of Hispanic patients presented emergently, compared with 13% of White patients. This pattern is consistent with upstream barriers such as limited access to cardiologists, inadequate screening, transportation difficulties, and medical mistrust [19, 20, 25–28]. Breathett et al found that Black heart failure patients were less likely to receive cardiologist care even in the intensive care unit (ICU) [19]. Patients presenting emergently have less opportunity for optimization and may have more advanced disease than severity scores reflect.

The divergent patterns across outcomes provide additional insight. Length of stay disparities were explained by measured covariates, indicating that differences reflected patient complexity rather than differential inpatient treatment. In contrast, charge disparities persisted, possibly reflecting regional pricing variation, complication rates not captured by mortality, or differences in ICU utilization.

The persistent elevation in mortality observed among Asian/Pacific Islander patients (adjusted OR, 2.20; 95% CI, 1.30–3.72) warrants attention but is difficult to interpret with confidence. NIS aggregates a highly heterogeneous population into a single category [29, 30]. This group includes South Asian, East Asian, Southeast Asian, Native Hawaiian, and Pacific Islander individuals, who have substantially different cardiovascular risk profiles, body-composition norms, and care-access patterns. With 846 patients in this combined group, further subgroup analysis was not feasible. This finding should be interpreted cautiously and motivates focused study in datasets with finer ethnic granularity.

The lack of temporal improvement over 7 years is discouraging. Despite expanded TEER availability and growing attention to health equity, mortality gaps have not narrowed. Targeted interventions may be necessary.

### Clinical implications

Structural heart programs should examine referral networks to ensure equitable access. Outreach to primary care providers in underserved areas may facilitate earlier identification and referral. Quality improvement efforts should stratify outcomes by race to monitor disparities. Perioperative optimization may help mitigate excess risk from late presentations. At the policy level, these data support investment in cardiovascular infrastructure serving minority communities and incorporation of equity metrics into value-based payment models.

### Strengths and limitations

Strengths include the large sample, nationally representative data, contemporary timeframe capturing TEER adoption, and comprehensive adjustment. Limitations include the administrative nature of NIS data, which lacks clinical detail such as echocardiographic parameters, functional status, and procedural specifics, and cannot distinguish primary from secondary MR [31, 32]. NIS does not provide hospital procedure volume directly, only bed-size and teaching status as proxies. Mitral valve surgery and TEER are concentrated in high-volume referral centers, and our cohort skews toward large hospitals (72%) and teaching centers (91%). Although sensitivity analyses restricted to large or teaching hospitals demonstrated that the procedural disparity persists in these subgroups (OR 1.33 and 1.38, respectively), we cannot assess hospital procedure-specific volume, surgeon volume, or the presence of a dedicated structural heart program, each of which may confound the relationship between race and procedure selection [24]. Future work using Society of Thoracic Surgeons (STS) or transcatheter valve therapy (TVT) registry data is needed to characterize the role of hospital infrastructure. Race is self-reported and subject to misclassification. We examined only inpatient outcomes; longer-term outcomes and functional recovery cannot be assessed [33, 34]. Residual confounding from unmeasured variables cannot be excluded.

### Conclusions

Among 26,456 patients undergoing mitral valve intervention in the United States from 2016 to 2022, Black patients were more likely to receive TEER than open repair across all sensitivity analyses (adjusted OR, 1.36; 95% CI, 1.15–1.61), a finding that persisted in high-volume and teaching centers and after race-balanced propensity weighting. Black patients also experienced higher in-hospital mortality, concentrated within the severe heart failure subgroup, whereas Asian/Pacific Islander patients had consistently elevated mortality across all models (adjusted OR 2.20). These disparities likely reflect a combination of differences in clinical presentation, comorbidity burden, referral patterns, and potentially procedural decision-making. Administrative data cannot definitively distinguish these mechanisms, and our findings should prompt prospective investigation in registries with surgical-risk and MR-etiology data.

## Data Availability

The data that support the findings of this study are available from HCUP at the Agency for Healthcare Research and Quality (AHRQ). Restrictions apply to the availability of these data, which were used under a Data Use Agreement for this study. The NIS is available to qualified researchers through HCUP at https://www.hcup-us.ahrq.gov/ upon completion of the HCUP Data Use Agreement training and payment of applicable access fees.

## Abbreviations

AF: atrial fibrillation
AHRQ: Agency for Healthcare Research and Quality
APR-DRG: All Patient Refined Diagnosis Related Group
CAD: coronary artery disease
CI: confidence interval
CKD: chronic kidney disease
COPD: chronic obstructive pulmonary disease
DUA: Data Use Agreement
ED: emergency department
ESS: effective sample size
HCUP: Healthcare Cost and Utilization Project
HF: heart failure
HTN: hypertension
ICD-10-CM: International Classification of Diseases, 10th Revision, Clinical Modification
ICD-10-PCS: International Classification of Diseases, 10th Revision, Procedure Coding System
IPTW: inverse probability of treatment weighting
IRB: Institutional Review Board
IRR: incidence rate ratio
LOS: length of stay
MR: mitral regurgitation
NIS: National Inpatient Sample
NS: not significant
OR: odds ratio
PI: Pacific Islander
PS: propensity score
PSW: propensity score weighting
SD: standard deviation
SMD: standardized mean difference
STS: Society of Thoracic Surgeons
TAVR: transcatheter aortic valve replacement
TEER: transcatheter edge-to-edge repair
TVT: transcatheter valve therapy (Registry)
